# Pathogenesis and comprehensive treatment strategies of sarcopenia in elderly patients with type 2 diabetes mellitus

**DOI:** 10.3389/fendo.2023.1263650

**Published:** 2024-01-08

**Authors:** Yang Hou, Jia Xiang, Bo Wang, Shoufeng Duan, Rouxuan Song, Wenhu Zhou, Songwen Tan, Binsheng He

**Affiliations:** ^1^Hunan Provincial Key Laboratory of the Research and Development of Novel Pharmaceutical Preparations, Changsha Medical University, Changsha, Hunan, China; ^2^Xiangya School of Pharmaceutical Sciences, Central South University, Changsha, Hunan, China

**Keywords:** sarcopenia, type 2 diabetes, elderly, pathogenesis, treatment

## Abstract

Sarcopenia and diabetes are two age-related diseases that are common in the elderly population, and have a serious effect on their general health and quality of life. Sarcopenia refers to the progressive loss of muscle mass, strength and function, whereas diabetes is a chronic disease characterized by elevated blood sugar levels. The comorbidity of sarcopenia and diabetes is particularly concerning, as people with diabetes have a higher risk of developing sarcopenia due to the combination of insulin resistance, chronic inflammation and reduced physical activity. In contrast, sarcopenia destroyed blood sugar control and exacerbated the development of people with diabetes, leading to the occurrence of a variety of complications. Fortunately, there are a number of effective treatment strategies for sarcopenia in people with diabetes. Physical exercise and a balanced diet with enough protein and nutrients have been proved to enhance the muscular quality and strength of this population. Additionally, pharmacological therapies and lifestyle changes can optimize blood sugar control, which can prevent further muscle loss and improve overall health outcomes. This review aims to summarize the pathogenesis and comprehensive treatment strategies of sarcopenia in elderly patients with type 2 diabetes, which help healthcare professionals recognize their intimate connection and provide a new vision for the treatment of diabetes and its complications in this population. Through early identification and comprehensive treatment, it is possible to improve the muscle function and general quality of life of elderly with diabetes and sarcopenia.

## Graphical Abstract

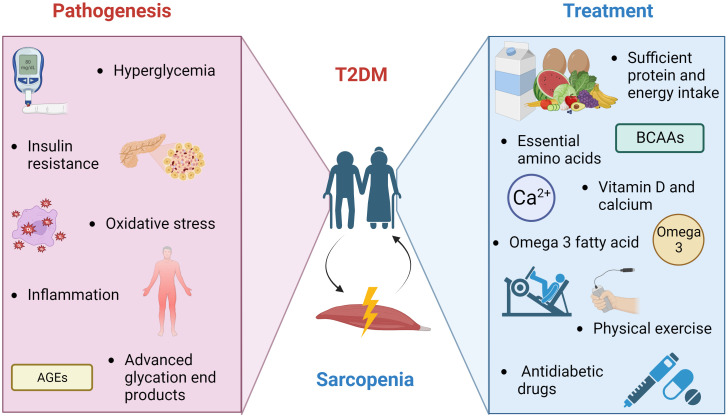

## 1 Introduction

Sarcopenia is defined as a disease characterized by progressive loss of skeletal muscle mass and function, which usually occurred in the elderly (1, 2). The loss of skeletal muscle mass and function seriously affect the physical health and quality of life of individuals, leading to difficulties in daily activities and increased risk of falls and fractures, even shortened life expectancy of the elderly (3). Risk factors leading to sarcopenia include age, unbalanced diet and physical inactivity. In addition, inflammation and chronic diseases may accelerate its development (4). In 2016, sarcopenia was officially recognized as a disease with distinctive characteristics by the medical community (5). The prevalence of sarcopenia is approximately 10% in individuals aged 60 and above, whereas it reaches 20% to 30% in those aged 80 and above (6, 7). In view of the aging trend of global population, sarcopenia has emerged as an important health concern. The global population aged 65 years or above is expected to increase from 9% in 2019 to nearly 17% in 2050, which increase the risk of sarcopenia (8).

Diabetes mellitus is a chronic metabolic disease, which is mainly characterized by elevated blood sugar levels (9, 10). More than 60% of individuals aged 65 and above have diabetes, while 50% of the elderly people have prediabetes (11). According to the estimation of the International Diabetes Federation, the number of people with diabetes will reach up to 783 million by 2045 (12). Type 2 diabetes mellitus (T2DM) is the most common type, accounting for more than 90% of people with diabetes (13–15). Persistent hyperglycemia can lead to a range of complications, which have a serious impact on the cardiovascular system, kidneys and eyes, thereby posing serious threats to the overall health (16–19). The complications and severity of diabetes lead to a considerable morbidity and mortality rate, make it one of the most urgent worldwide public health problems (20).

Sarcopenia and diabetes in the elderly are intricately linked and have a negative impact on each other (21). Sayani Das examines the relationship between diabetes and sarcopenia in India’s elderly population revealed that individuals with diabetes had a significantly higher risk of sarcopenia (22). Moreover, recent meta-analyses have revealed that the prevalence of sarcopenia is two to three times greater among patients with diabetes in comparison to those without the condition (23, 24). Osaka et al.’s study further underscored the connection by indicating that sarcopenia elevates the risk of nephropathy in people with diabetes (25). According to Feng et al.’ study, the pooled prevalence of sarcopenia in people with diabetes reached up to 18%, which has become an urgent situation to be prevented early (26). The purpose of this paper is to review the pathogenesis and comprehensive treatment strategies of sarcopenia in patients with T2DM, which aims to help clinicians and nutritionists to identify and intervene at an early stage, ultimately enhancing the overall health and well-being of these patients.

## 2 Sarcopenia

### 2.1 Definition and prevalence of sarcopenia

In 1989, the term sarcopenia was initially coined by Rosenberg in 1989 (27, 28). The European Working Group on Sarcopenia in the Elderly (EWGSOP) first defined sarcopenia in 2010 as “age-related, generalized decrease in muscle mass and/or decrease in muscle strength or muscle physiology” (29). A similar agreement was subsequently announced in 2014 by the Asian Sarcopenia Working Group, who also altered the pertinent diagnostic cutoff for the Asian population (30). Sarcopenia was recognized as a disease on a global scale and included in the ICD-10 (M62.84) in 2016 (5). The European Sarcopenia Working Group presented a revised definition of sarcopenia in 2018 and an explanation of muscular function. Sarcopenia was defined as the gradual loss of muscle mass, strength, and muscle function (31). It is difficult to determine the prevalence of sarcopenia consistently since different cutoffs and measurement methods result in dramatically different incidences of the disease across studies. According to a meta-analysis of studies, the prevalence in adults over 60 ranges from 10 to 27 percent (32). The number of sarcopenia patients worldwide will rise year after year as the population ages.

### 2.2 Diagnosis process of sarcopenia

**Figure 1:**
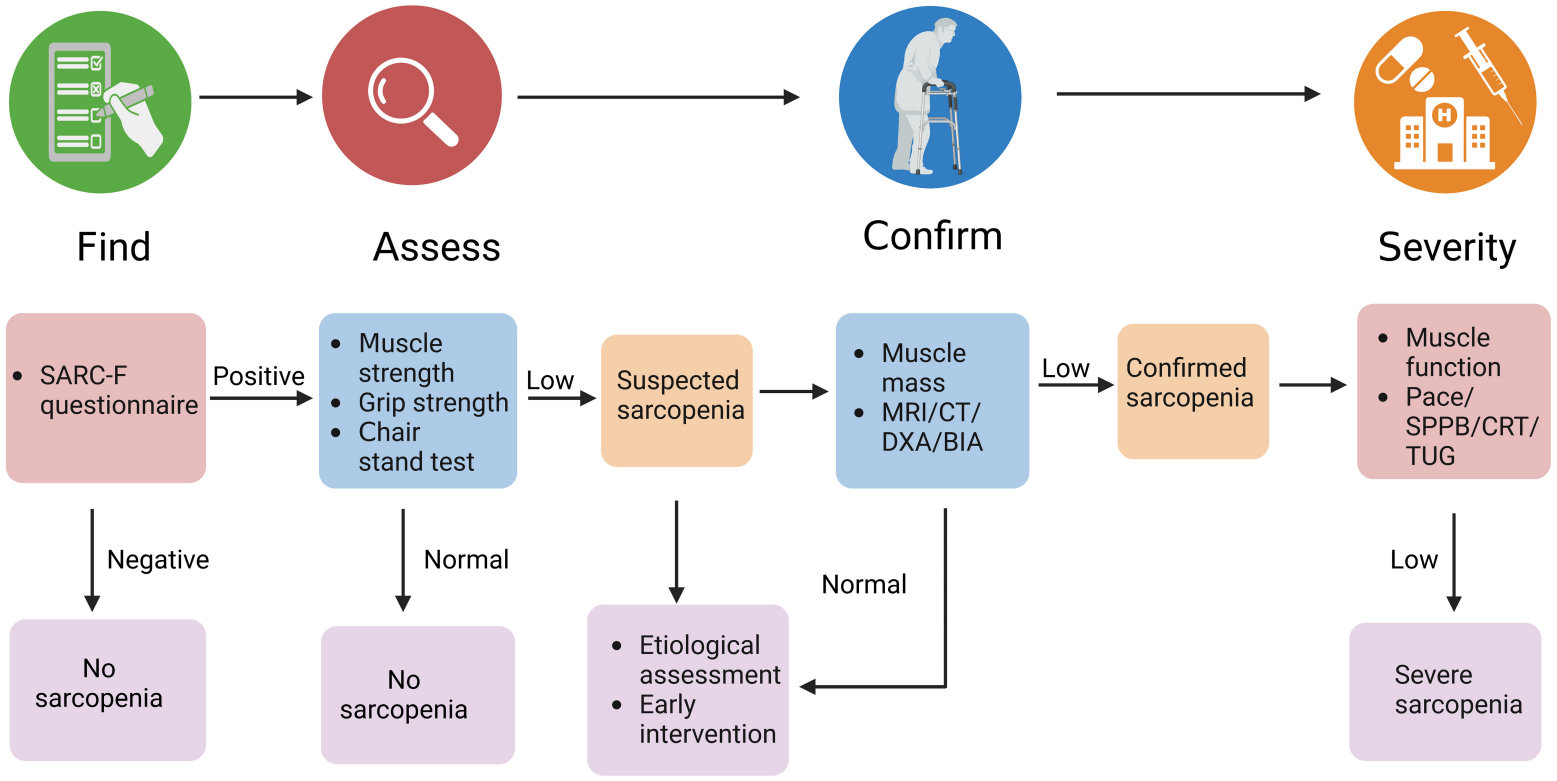
The diagnostic process for sarcopenia.

The diagnostic process for sarcopenia was updated by the European Working Group on Sarcopenia in the Elderly as Find-Assess-Confirm-Severity, as illustrates in **Figure 1**, noting that sarcopenia is considered severe when low muscle strength, low muscle mass and low physical function are all detected (31).

#### 2.2.1 SARC-F questionnaire

The European Working Group on Sarcopenia in the Elderly recommends using the SARC-F questionnaire to assess sarcopenia risk. The SARC-F is a simple five-item self-assessment questionnaire created by Morely (33). SARC-F is a rapid and simple screening method for sarcopenia in the elderly, which has been used widely used worldwide (34). Strength, assisted walking, getting up, climbing stairs, and falling are all assessed. A sarcopenia risk is present if the sum of the scores for each of the five items is greater than or equal to 4 points providing that the maximum total score is 10 points (35). However, due to the high specificity and relatively low to moderate sensitivity of SARC-F in predicting sarcopenia, many patients with sarcopenia may go misjudged (36–39). As a result, the SARC-CalF approach has been proposed, which adds a maximum leg circumference based on SARC-F. The results of several researches indicated that this method significantly improves the sensitivity and accuracy of evaluation (40, 41).

#### 2.2.2 Muscle strength

Muscle strength is the maximal force that one or more muscular groups may generate. The European Working Group on Sarcopenia in the Elderly regards muscle strength as the main reference index for sarcopenia. It is recommended to utilize grip strength as an additional measure when assessing muscular strength because it has a relatively positive correlation in other body sections and is simple to use (42, 43). Upper limb muscle grip strength, which can be measured by a manual ergometer, is typically used to assess grip strength in the upper limbs. The chair stand test measures how quickly patients can stand up from the chair without the aid of their arms, and to assess the muscle strength of their lower limbs (31).

#### 2.2.3 Muscle mass

Muscle mass refers to the total amount of skeletal muscle in the body. Computed tomography (CT), magnetic resonance imaging (MRI), dual-energy X-ray absorptiometry (DXA), and bioelectrical impedance analysis (BIA) are common used to estimate the skeletal muscle mass (31). CT and MRI are the current gold standard for assessing muscle mass with high accuracy and reproducibility. Their use is restricted due to their high price, complexity, and high radiation exposure from CT (44). DXA is considered to be one of the ideal methods for determining muscle mass with its quick operation and minimal radiation exposure (45). However, DXA equipment cannot be widely used in hospitals and communities since it is difficult to carry. Instead, due to its simplicity, portability, and affordability, BIA is better suited for extensive hospitals and communities screening and can be used as a less expensive alternative to assess muscle mass (29, 44, 45).

#### 2.2.4 Muscle function

Muscle function is defined as objectively measured exercise-related systemic function. Its testing methods include daily pace assessment, short-physical performance battery (SPPB), the chair rising test (CRT), and stand-up and go test (Time-up and go test, TUG). Daily Gait speed is a quick, secure and reliable sarcopenia test, which can indicate the health status and is related to their survival possibility of the elderly (46). A walking speed of less than 0.8 m/s is suggested by EWGSOP2 as a sign of severe sarcopenia (31). SPPB is a compound test method of muscle function, which is a standard method in both research and clinical application and is widely used (47, 48). Three tests are included in SPPB, with the highest score of 4 points and the full score of 12 points for each individual test. The risk of poor muscle function is indicated by a score of 9 or less.

### 2.3 Risk factors of sarcopenia

#### 2.3.1 Intrinsic factors

**Figure 2:**
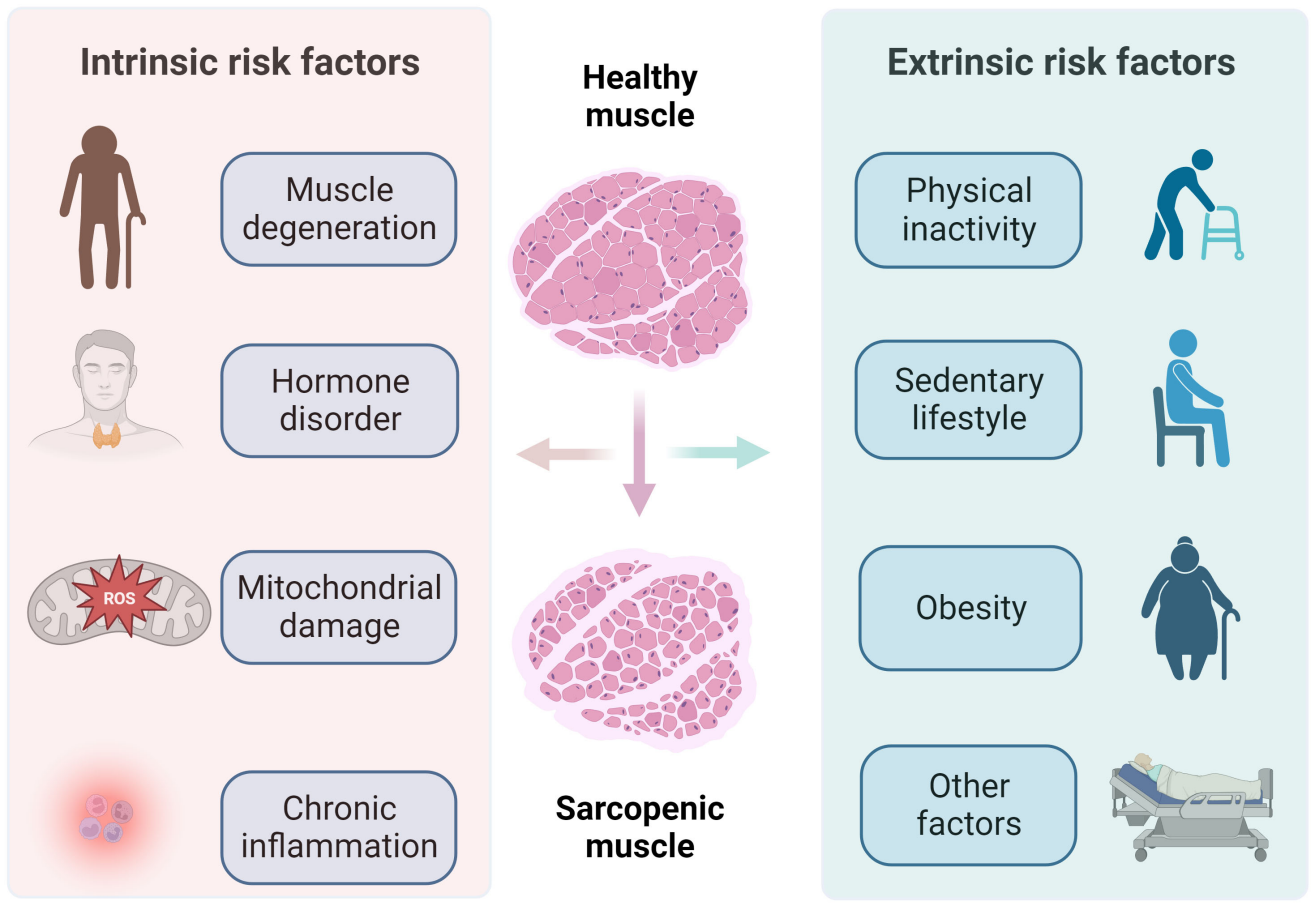
Intrinsic and extrinsic risk factors of sarcopenia.

Sarcopenia is a complicated and multifactorial process, which is influenced by both intrinsic and extrinsic factors, as depicted in **Figure 2**. Age is considered as a key intrinsic risk factor of sarcopenia. Because age not only directly affects muscle quality and function, but also indirectly increases the risk of sarcopenia by bringing some problems such as hormone disorder, mitochondrial damage and chronic inflammation. With the increase of individual age, the metabolic rate of human body gradually decreases. This metabolic leads to the inhibition of protein synthesis, thereby impairing muscle repair and regeneration capabilities (1). Age-related muscle tissue alterations include serious loss of muscle mass, modifications in muscle fiber morphology, deterioration of muscle function, and reduction of muscle contraction time and strength. Consequently, sarcopenia is widely regarded as an age-related disease (49). Furthermore, age also affects the structure and function of skeletal muscle fibers. As age advances, there is a gradual decrease in the quantity and size of muscle fibers with the skeletal muscle, along with an increase in the presence of fat and connective tissue within the muscle matrix, all of which contribute to the degradation of muscle mass (50). Therefore, it can be seen that age is an important factor of sarcopenia, which affects the health and function of muscles in many ways.

Moreover, the imbalance of the human endocrine system and the fluctuation of hormone levels caused by aging contribute to an increased risk of sarcopenia by affecting skeletal muscle function. An obvious example is that with adult man ages, the decrease of muscle mass and strength is closely related to steady decline testosterone levels (51). As skeletal muscle undergoes the aging process, the level of reactive oxygen species (ROS) gradually increases, disrupting the body’s ability to maintain oxidative balance. This disruption decreases myogenic differentiation and triggers regenerative errors during the skeletal muscle degeneration (52–55). ROS are mainly produced in mitochondria, which also serve as the primary target of intracellular oxidative stress. High levels of ROS can damage mitochondria, resulting in abnormal mitochondrial function, and potentially contributing to the development of sarcopenia (56–58). In addition, the proliferative and differentiation capabilities of satellite cells decline significantly with age, compromising the regenerative potential of muscle cells. These changes result in fibrosis of aging muscle, thereby considered as a pivotal factor in the initiation of sarcopenia (59–61).

Chronic inflammation related to aging is another important risk factor for sarcopenia (62). The increase of pro-inflammatory cytokine levels and the decrease of anti-inflammatory cytokine levels *in vivo* are indicators to assess chronic inflammation. Loss of muscle mass and strength is associated with an increase in inflammatory markers in the blood, which reduces lower limb function and physical activity (63, 64). According to several horizontal and longitudinal studies, sarcopenia is associated with high levels of proinflammatory cytokines, including tumor necrosis factor-α (TNF-α), interleukin-6 (IL-6), and C-reactive protein (CRP). These inflammatory factors are secreted by inflammatory and immune cells, which inhibit muscle synthesis and promote muscle degradation, consequently result in sarcopenia (65–67).

#### 2.3.2 Extrinsic factors

The decline of exercise ability are the main reasons for the loss of muscle mass and strength in the elderly (68). Muscle tissue is highly plastic, which needs constant participation and stimulation under load to maintain the proper metabolism and repair functions. Lack of exercise will result in insufficient muscle stimulation, deceleration of protein synthesis, and decrease of muscle mass (69). Moreover, fear of falling causes psychological burden to the elderly, which makes them unwilling to do exercise and increases the risk of muscle loss. At the same time, physical inactivity can easily lead to obesity in the elderly. The weight of the elderly mostly increases in the form of fat rather than thin tissue, and the occurrence of sarcopenia is usually accompanied by the growth of adipose tissue, which promotes each other (70).

In addition to physical inactivity, it is worthing noting that the elderly who have been engaged in a sedentary lifestyle for a long time face a higher risk of sarcopenia. When sedentary time exceeds 2 hours, even physically active elderly people are prone to sarcopenia, which emphasizes that physical activity itself cannot offset the harmful consequences of long-term inactivity (71). Although muscle mass and muscle fibers will naturally decrease with age, the risk of sarcopenia can be effectively reduced by performing moderate to high-intensity physical activities instead of sitting for a long time (72).

Other extrinsic factors such as muscle disuse caused by physical trauma, chronic stress and long sleep will increase the risk of muscle loss (73–75). In addition, long-term exposure to certain pollutants or toxins will adversely affect muscle health and overall health. Extreme weather conditions or geographical location may affect individuals’ ability to engage in outdoor activities and obtain fresh food, thus increasing the risk of sarcopenia. It must be recognized that the progress of sarcopenia is a dynamic process, and the early symptoms are often difficult to find, which leads to the neglect of this disease. In fact, it is very common for most elderly people to take this problem seriously only when pathological conditions and adverse consequences appear. Therefore, it is very important to pay attention to sarcopenia in the elderly at an early stage.

## 3 Pathogenesis of interaction between sarcopenia and T2DM

### 3.1 Insulin resistance and muscle mass

Insulin resistance is considered as the main pathophysiological basis of T2DM, which plays an important role in the occurrence and development of sarcopenia (76–78). Insulin is very important to control metabolism, and its main function is to promote protein synthesis, muscle growth and the absorption and utilization of glucose by muscle tissue (79). Insulin resistance occurs when the sensitivity and reactivity of insulin receptors decrease. As a result, blood sugar fails to effectively enter muscle tissue. This leads to insufficient nutrition supply of muscle tissue, which leads to muscle atrophy and reduction (80). Skeletal muscle can absorb 75% of blood sugar through insulin signaling system (81). Therefore, muscle cells with insulin signaling system problems will not be able to effectively use the glucose in the blood stream, leading to accelerated protein decomposition and accelerated muscle loss (82–84). Low muscle mass may make blood sugar control disorder, further aggravating the development of diabetes (78).

### 3.2 Hyperglycemia

Hyperglycemia is a significant risk factor leading to the loss of muscle mass and function during aging (85), which affect the development and progress of sarcopenia through various mechanisms. Firstly, hyperglycemia leads to an increase in the formation of advanced glycation end products, which accumulate in muscle tissue, destroy muscle contraction and endothelial function, and finally worsen muscle function (86). Secondly, the oxidative stress and inflammatory reaction caused by hyperglycemia will lead to muscle cell damage and cell death (87, 88). In addition, the increase of blood sugar level declines the responsiveness of muscle cells to insulin, which reduces the absorption and utilization of glucose and amino acids by these cells. Muscle tissue cannot get enough nutritional support, leading to the aggravation of sarcopenia (89). Lastly, hyperglycemia affects the energy metabolism of muscle cells by destroying the balance between glycogen production and decomposition. This disorder leads to the decrease of glycogen storage in muscle cells, which leads to the insufficient energy supply of these cells and accelerates the onset of sarcopenia (90). In a word, hyperglycemia poses many threats to muscle health in the process of aging, affecting the structure and function of muscle tissue.

### 3.3 Oxidative stress and inflammatory reaction

Muscle atrophy in people with diabetes mainly comes from oxidative stress and inflammation. Chronic inflammation, oxidative stress, insulin resistance, the formation of advanced glycation end products and hyperglycemia are all linked to cellular redox imbalance (88, 91). This factors together hinder the metabolism of protein in skeletal muscle, leading to increased oxidative damage of DNA, lipids and protein (92). The increase of lipid metabolites in patients with T2DM can lead to excessive production of ROS. This overproduction can be alleviated by antioxidant enzymes, which play a vital role in regulating muscle regeneration, inducing angiogenesis and reducing muscle fibrosis. Furthermore, the high level of ROS will reduce muscle fiber size and muscle mass, destroy muscle homeostasis, interfere with muscle production, and thus aggravate muscle atrophy (93). Inflammatory response is a complex and important biological mechanism, which responds to pathogens and injury stimuli by activating immune cells and releasing inflammatory mediators, thus protecting the body against injury (94). However, persistent chronic inflammation may represent a potential pathological condition that damages muscle tissue and promote the development and progress of sarcopenia (95).

### 3.4 Sarcopenic obesity

Sarcopenic obesity (SO) is defined as the co-existence of obesity and sarcopenia in a consensus achieved by the European Society for Clinical Nutrition and Metabolism and the European Association for the Study of Obesity (ESPEN-EASO) in 2022 (96). The prevalence of sarcopenic obesity is high among the elderly, with about one in every ten people suffering from it (97). A large number of studies have shown that SO can increase more negative effects than sarcopenia or obesity alone, including a significant increase in the probability of falling, disability (98). Obesity exists in both people with sarcopenia and diabetes. A study shows that more than half of diabetics are obese at the same time (99). Therefore, the coexistence of SO and T2D has become a common health problem. Studies have shown that the prevalence of SO in diabetic patients is as high as 27%, and it is related to serious adverse consequences (100). Obesity and T2D are one of the main causes of the dysfunction of skeletal muscle stem cell regeneration, which is manifested by the loss of muscle content and the progressive decline of glucose and lipid metabolism of skeletal muscle, thus accelerating the process of diabetes and entering a vicious circle (101). Therefore, we should pay attention to the early screening and identification of SO in people with diabetes, and choose appropriate intervention measures to reduce the incidence and various adverse outcomes in this population.

## 4 Comprehensive treatment strategies

### 4.1 Control of blood sugar level

Diabetes is a chronic disease characterized by elevated blood sugar level, which can lead to the occurrence of various complications. Among these, muscle loss, known as sarcopenia, is a common complication influenced by high blood sugar. The decrease of mitochondrial activity induced by hyperglycemia aggravates insulin resistance and impairs energy metabolism (102). Furthermore, the inflammatory responses triggered by hyperglycemia will further lead to insulin resistance and muscle atrophy by inhibiting insulin signaling (103). Therefore, blood sugar control is crucial for people with diabetes and sarcopenia. Effective management of blood sugar level can slow down the rate of muscle loss and reduce protein decomposition. In well-controlled people with diabetes, stable insulin level contributes to the synthesis of muscle protein and promotes muscle quality and strength. This can not only prevent the onset of sarcopenia, but also accelerate muscle growth and repair, and ultimately enhance the overall muscle quality and body function. Skeletal muscle, as the most important exercise organ of the human body, assist in storing protein, regulating glucose metabolism and stabilizing blood sugar level, contributing to overall health of the body (81, 104, 105).

### 4.2 Nutrition intervention

Compared with the general population, people with diabetes face a higher risk of malnutrition and are more prone to sarcopenia. Nutritional therapies have the potential to prevent or even reverse sarcopenia and frailty (106). Two elderly patients with malignant hematological diseases were studied by Matsunaga et al., and the effectiveness of rehabilitation treatment on sarcopenia was reported (107). Chan et al.’s study noted that expanding nutritional counseling for people with diabetes and effectively reduce the incidence of sarcopenia (108). Several studies demonstrate that diet is no obvious evident benefit for elderly with diabetes and sarcopenia (102, 103, 109). However, the majorities of studies have consistently shown the positive effects of diet and nutrition intervention in the prevention and control of sarcopenia (110–119). Moreover, for many elderly people who cannot exercise, nutritional intervention is still the most promising treatment and prevention strategy (120, 121). Therefore, it is crucial to emphasize the importance of a balanced diet, comprehensive nutrition and vigilant monitoring of diabetes and malnutrition in the elderly. The comprehensive overview of the relationship between nutritional intervention and sarcopenia is shown in **Table 1**.

**Table 1:** Studies on the effect of nutritional intervention on sarcopenia in elderly people with diabetes.

Nutrition intervention	Study design	Target population	Age (years old)	Research objectives	Results	References
Protein intake	Cross-sectional	T2DM patients	65.0 ± 7.7	Physical function outcomes	Positively associated	(110)
Protein intake	Cross-sectional	T2DM patients	≥31	Muscle loss	Negatively affected	(111)
Nutrition counseling	Cross-sectional	T2DM patients	45-90	Sarcopenia	Negatively associated	(108)
Mediterranean Diet	Cross-sectional	Overweight or Obese T2DM patients	71.2 ± 8.2	Gait speed	Positively associated	(112)
Energy intake	Cross-sectional	T2DM patients	≥65	The presence of sarcopenia	Negatively associated	(113)
Vitamin D intake	Prospective cohort study	T2DM patients	≥65	Muscle loss	Negatively associated	(114)
Omega-3 fatty acids intake	Cross-sectional	T2DM patients	74.2 ± 5.7	Sarcopenia	Negatively associated	(115)
Malnutrition	Cross-sectional	T2DM patients	76.6 ± 6.27	The prevalence of sarcopenia	Negatively associated	(116)
Resistance training and nutritional interventions	Prospective cohort study.	Frail T2DM patients	79 ± 5.6	SPPB, maximal strength and power output	Positively improved	(117)
Nutrition intervention	Randomized controlled trial	Obese and overweight patients with diabetic foot ulcers	30-70	Body composition	No significant correlation	(102)
Vitamin D intake	Randomized controlled trial	Obese T2DM patients	30-60	Body composition	No significant correlation	(103)
Branched chain amino acids	Longitudinal study	T2DM patients	56.6 ± 10.6	Skeletal muscle loss	Negatively associated	(118)
Serum vitamin D level	Prospective observational cohort study	Men	59.1 ± 10.5	Diabetes	No significant correlation	(109)
Calorie-restricted diet and recreational sports training	prospective study	T2DM patients	60.0 ± 6.0	Expression of skeletal muscle gene	Downregulates the expression of atrophy-associated myokines and increases the expression of anti-inflammatory gene IL-15	(119)

#### 4.2.1 Protein

Protein intake is beneficial to the elderly people with both sarcopenia and T2DM. Increasing protein’s habitual intake can not only effectively improve blood sugar control and maintain muscle mass, but also promote weight control, reduce inflammation and increase insulin sensitivity (122). Protein is a key basic element of muscle function and an indispensable nutrient for muscle synthesis. Wu et al. revealed that the increase of protein intake was related to the increase of grip strength and the decrease of prevalence of sarcopenia (123). Many studies have consistently confirmed that adequate intake of protein can not only stimulate the synthesis of protein in muscle, but also reduce the degradation process of protein and improve the effective utilization of nutrients in muscle (124–126). In addition, proper protein intake has a positive effect on blood sugar regulation, which helps to reduce the risk of diabetes. Protein intake can directly promote postprandial insulin secretion, which may be beneficial to the blood sugar regulation of elderly T2D patients (127). Studies have pointed out that the elderly with sarcopenia should consume 1.6g/kg of protein every day, and the ESPEN expert group and the PROT-AGE research group also suggested that people with chronic diseases (such as diabetes) should consume about 1.2-1.5g/kg/day of protein (128–130). Therefore, higher protein intake should be encouraged for the elderly people with sarcopenia and T2DM.

#### 4.2.2 Essential amino acids

Amino acids are the basic units of muscle protein, and the impact of protein consumption on the rate of muscle synthesis mainly focus on essential amino acids (131, 132). These essential amino acids play a vital role in both muscle synthesis and maintenance, demonstrating a linear relationship with muscle protein synthesis within a specific dosage range (132). In particular, leucine stands out because of its remarkable stimulating effect (133–135). Long-term continuous intake of essential amino acids rich in leucine is related to significant enhancement of skeletal muscle mass, strength, and walking speed in the elderly (136). Rieu et al. studied the completely balanced diet of 20 healthy male subjects with or without leucine, and found that leucine is effective in promoting the synthesis of muscle protein and counteracting the influence of sarcopenia (137). A study emphasizes that leucine can act as insulin secretagogue to effectively improve postprandial blood glucose control and promote the synthesis of muscle protein (138). Therefore, ensuring adequate dietary intake of protein and essential amino acids has become a key factor to maintain overall health and function.

#### 4.2.3 Vitamin D and calcium

Vitamin D play an important role in maintaining muscle health and has a direct impact on muscle development. Vitamin D is a fat-soluble nutrient that strengthens bone health by promoting the absorption of essential minerals such as calcium and phosphorus. A paired case-control study by Yang et al. showed that vitamin D deficiency was associated with an increased risk of sarcopenia (139). Compared with young women with sufficient vitamin D level, young women with insufficient vitamin D level showed a decrease in muscle mass, and at the same time, the fat penetration in muscle increased by about 24%, further highlighting the far-reaching influence of vitamin D on muscle strength (140). In addition, compared with people with sufficient vitamin D, the grip strength of the elderly with low serum vitamin D level is significantly reduced (141). Visser et al. conducted a longitudinal aging study and found that vitamin D deficiency is not only associated with the weakening of muscle function and the onset of sarcopenia, but also increases the risk of osteoporosis and fracture (142). Individuals with sarcopenia consume lesser essential nutrients such as calcium, iron and zinc than the general population, further aggravating the problems related to muscle health (143). Encouragingly, studies involving elderly women show that continuous supplementation of vitamin D and calcium can significantly improve skeletal muscle performance and reduce the incidence of falls (144). These comprehensive studies emphasize the potential benefits of vitamin D and calcium supplements in promoting muscle health, especially in the elderly and individuals with chronic diseases.

#### 4.2.4 Omega-3 fatty acids

A growing body of evidences emphasize the potential role of omega-3 fatty acids in preserving and regulating the quality and function of skeletal muscle. Robinson et al.’ study on diet and its relationship to grip strength in community-dwelling older men and women revealed a positive correlation between eating fatty fish and increasing grip strength (145). Dos Reis et al.’s study revealed that the intake of omega-3 fatty acids is negatively correlated with the incidence of sarcopenia, and a favorable relationship with the skeletal muscle mass index of limbs was also proved (146). A systematic meta-analysis conducted by Bird et al. further supported these findings, emphasizing the positive effects of supplementation of omega-3 long-chain polyunsaturated fatty acids on muscle quality and strength (147). A study showed that omega-3 fatty acid supplements are also expected to reduce inflammation (148). Rats fed with a combination of omega-3 polyunsaturated fatty acids derived from fish oil and wheat oligopeptides showed significant potential to prevent age-related muscle loss and reduced oxidative stress and inflammation in skeletal muscle (149). The comprehensive findings of these studies demonstrate that omega-3 fatty acids have positive and multifaceted potential in muscle protection and inflammation reduction, and are expected to become a potential nutritional support strategy for elderly patients with sarcopenia.

### 4.3 Physical exercise

Physical exercise is considered as a safe and efficient intervention measures to prevent and treat diabetes mellitus complicated with sarcopenia. Participating in any form of physical activity can significantly improve sarcopenia in the elderly, which has been supported by a lot of research (116, 150–157), as shown in **Table 2**. On a global scale, physical exercise occupies a central position as the main prevention and treatment method of sarcopenia (31). Studies from different countries have consistently emphasized the far-reaching advantages of regular physical activity in maintaining muscle quality and function (158–162). Studies have emphasized the dual benefits of aerobic exercise and anaerobic exercise in reducing insulin resistance. Aerobic exercise can especially enhance insulin sensitivity, while resistance training and strength training can also significantly improve insulin resistance (163, 164). For those elderly who struggle with sarcopenia, physical exercise, with special emphasis on resistance training, is considered as an effective strategy to combat the decline of activity ability related to age-related chronic diseases (165–168). When combined with nutritional supplements, resistance training can maximize its intervention (169, 170). According to international guidelines, it is recommended that patients with T2DM participate in resistance training at least twice a week (171). Therefore, it is imperative to incorporate resistance training into the exercise guide tailored for the elderly.

**Table 2:** Studies on the effect of physical exercise on sarcopenia in elderly people with diabetes.

Physical exercise intervention	Duration	Target population	Age (years old)	Research objectives	Results	References
Physical exercise	/	T2DM patients	76.6 ± 6.27	The prevalence of sarcopenia	Negatively associated	(116)
Short-term acute moderate-intensity resistance exercise	24h	Older T2DM patients	/	Blood glucose	Blood glucose decreased	(150)
Progressive Sandbag Exercise Training	12 weeks	T2DM and possible sarcopenia patients	>50	Muscle strength	Significantly improved	(151)
Circuit resistance training	10 weeks	T2DM patients	>65	Physical and metabolic function	Effective	(152)
Muscle power training	12 weeks	T2DM patients	70.5 ± 7.8	Functional capacity, body balance and lower limb muscle strength	Significantly improved	(153)
Resistance training	12 weeks	T2DM patients	69.7 ± 6.9	Lower limb strength and muscle mass	Significantly improved	(154)
Resistance training	12 weeks	T2DM patients	>65	Muscle function	Significantly improved	(155)
Low-volume walking high-intensity interval training (HIIT)	12 weeks	Older women with T2DM	/	Physical capacity	Significantly improved	(156)

### 4.4 Selection of antidiabetic drugs

The effect of antidiabetic drugs on sarcopenia is still ongoing research topic, with certain drugs demonstrating unclear effects. Some antidiabetic drugs, particularly long-term use of sulfonylurea drugs, such as glinide, gliclazide and glibedi, have been associated with muscle loss or injury. These drugs play a role by inhibiting KATP channel to stimulate insulin secretion (172). According to a large number of clinical reports, this mechanism is related to rapid skeletal muscle atrophy (173–175). On the contrary, some antidiabetic drugs show promise in maintaining muscle quality. In a study by Lee et al., it was observed that elderly men with diabetes experienced rapid skeletal muscle loss, but those who used insulin sensitizers such as metformin or thiazolidinedione lost significantly less weight than those who did not receive such treatment. This indicates that insulin sensitizer may weaken muscle loss (176). Some other studies have also shown that insulin administration can increase the skeletal muscle mass or gait speed of patients with diabetes, which may slow down sarcopenia in patients with T2DM (177–179). However, it is impossible to overlook the weight gain caused by insulin use (180). In conclusion, the effect of antidiabetic drugs on sarcopenia varies with specific drugs and individual conditions. Further clinical research is needed to fully clarify the complex relationship between antidiabetic drugs and sarcopenia.

## 5 Conclusion

In this study, the pathogenesis of sarcopenia in people with diabetes and comprehensive treatment strategies were summarized. There is a complex interrelationship between the two diseases. Type 2 diabetes leads to the progress of sarcopenia, and conversely, sarcopenia exacerbates the severity of diabetes. Lifestyle choice, age-related metabolic changes, elevated blood sugar levels and insulin resistance all contribute to sarcopenia in patients with T2DM. Therefore, healthcare professionals need to pay more attention to the early prevention of muscle loss in elderly diabetes patients.

Effective prevention and treatment of sarcopenia complicated by diabetes requires dietary intervention rich in protein, essential amino acids, vitamin D, calcium and other important nutrients (including omega-3 fatty acids). In addition, it is very necessary to advocate moderate exercise and physical exercise among patients with T2DM and sarcopenia. Considering all kinds of exercise types and intensities, it is very important to customize these activities according to the needs of the elderly. Equally important is the management of blood sugar level and the improvement of insulin resistance. When prescribing antidiabetic drugs, clinicians should include sarcopenia in the evaluation of its pharmacological effects. However, there is no specific drug for sarcopenia at present, which emphasizes the importance of active prevention.

Age-related sarcopenia has become an urgent global health challenge as the population ages. Diabetes is still one of the most common chronic diseases among the elderly. The complicated interaction and common pathogenic mechanism between these two diseases have a far-reaching impact on the health of the elderly. Health care professionals must give priority to the early detection, screening and diagnosis of sarcopenia in people with diabetes, and formulate comprehensive and reasonable preventive measures in time, and ultimately promote the well-being of the elderly population.

## Author contributions

YH: Writing – original draft, Conceptualization, Visualization, Writing – review & editing. JX: Writing – review & editing. BW: Writing – review & editing. SD: Writing – review & editing. RS: Writing – review & editing. WZ: Supervision, Writing – review & editing. ST: Supervision, Writing – review & editing. BH: Supervision, Writing – review & editing.
